# Intolerance of uncertainty and attitudes toward cancer as predictors of spiritual well-being in patients with cancer

**DOI:** 10.1007/s00520-026-10606-0

**Published:** 2026-03-27

**Authors:** Ebru Vatansever, Emre Ciydem

**Affiliations:** 1 Department of Nursing, Institute of Graduate Studies, Bandırma Onyedi Eylul University, Balıkesir, Türkiye; 2 Department of Mental Health and Psychiatric Nursing, Faculty of Nursing, Istanbul University, Istanbul, Türkiye

**Keywords:** Intolerance of uncertainty, Attitudes toward cancer, Spiritual well-being, Cancer, Stigma

## Abstract

**Purpose:**

This study aimed to explore the predictive roles of intolerance of uncertainty and attitudes toward cancer in determining the level of spiritual well-being among patients with cancer.

**Methods:**

The study population consisted of all patients diagnosed with cancer admitted to the oncology outpatient clinics of two different public hospitals or receiving inpatient treatment between March 1, 2024, and January 30, 2025. The sample consisted of 400 patients who met the inclusion criteria. In this study, three standardized instruments were used to assess the main study variables: the Three-Factor Spiritual Well-Being Scale (TFSWS), the Questionnaire for the Measurement of Attitudes Toward Cancer–Patient Version (QMATC-PV), and the Intolerance of Uncertainty Scale (IUS-12). Multiple linear regression analysis was used to analyze the data.

**Results:**

The mean scores of spiritual well-being, attitudes toward cancer, and intolerance of uncertainty were 118.63 ± 16.49, 2.06 ± 0.50, and 36.45 ± 9.80, respectively. There was a significant negative relationship between the impossibility of recovery and discrimination and spiritual well-being. There was a significant negative relationship between inhibitory anxiety and spiritual well-being. Furthermore, alcohol use and hormone therapy were significant variables for predicting spiritual well-being.

**Conclusion:**

The results of this study revealed that in addition to alcohol use and hormone therapy, the impossibility of recovery, discrimination, and inhibitory anxiety significantly affect spiritual well-being in cancer patients. These findings highlight the importance of addressing patients’ emotional and existential concerns as part of holistic cancer care. Interventions aimed at reducing uncertainty and improving attitudes toward cancer may enhance patients’ spiritual well-being and overall quality of life.

**Supplementary Information:**

The online version contains supplementary material available at 10.1007/s00520-026-10606-0.

## Introduction

Cancer is one of the most important health problems worldwide and has high mortality and morbidity rates. Approximately 20 million new cancer cases were reported globally in 2022 [1]. Furthermore, 9.7 million people were reported to have lost their lives due to cancer in the same year, and 1 in 8 women and 1 in 9 men worldwide were diagnosed with cancer during their lifetime [1]. Today, cancer is considered a global public health problem that affects individuals of all ages, increasing in frequency and strongly influencing many areas of their lives [2]. It is a disease process that not only threatens life but also triggers an existential crisis, leading individuals to question their values, belief systems, self-perception, and meaning in life [3, 4]. Following diagnosis, individuals face a long and uncertain treatment process characterized by the risk of relapse, concerns about life expectancy, and difficulties in coping with the disease [5, 6]. Thus, cancer deeply affects individuals at physical, psychological, social, and spiritual levels [7–10].

Spiritual well-being is a multidimensional concept that defines the capacity of an individual to give meaning to life, find inner peace, and establish harmony between oneself and the environment, which plays an important role in maintaining psychological and existential integrity in life-threatening disease processes [11, 12]. High levels of spiritual well-being increase patients’ quality of life, reduce death anxiety, and contribute to positive attitudes toward treatment [11, 13]. However, studies indicate that spiritual well-being may either strengthen or weaken under traumatic experiences depending on psychological and cognitive reactions and that certain personality or perception traits play a determining role [11, 14]. One of these key traits is intolerance of uncertainty.

Intolerance of uncertainty is defined as the tendency to perceive uncertain, unpredictable, or uncontrollable situations as distressing, threatening, or stressful, leading to intense anxiety or avoidance behaviors [5, 15]. Uncertainty in cancer patients is considerably high due to the unpredictability of the diagnosis and treatment process, the risk of recurrence, and uncertainties regarding life expectancy [5, 6, 16]. Consequently, the level of intolerance of uncertainty becomes critical for cancer patients. Previous studies have shown that cancer patients generally exhibit moderate to high levels of intolerance of uncertainty [17–20]. Individuals who struggle to cope with uncertainty may have difficulty finding meaning and inner peace, which negatively affects their spiritual well-being [21–23]. However, existing studies exploring this relationship have focused mostly on populations such as older adults or kidney transplant recipients [21–23]. Considering that cancer patients also experience high levels of uncertainty and are spiritually vulnerable [17, 18], the lack of studies directly examining the impact of intolerance of uncertainty on spiritual well-being among this group represents a significant gap in the literature.

Another important factor influencing spiritual well-being is individuals’ attitudes toward cancer, particularly in relation to stigma. Stigmatization refers to being perceived as defective, inferior, or vulnerable to social exclusion due to an individual’s characteristics, such as having a cancer diagnosis [24, 25]. In this context, three types of stigma can be distinguished: public stigma, referring to negative societal attitudes toward people with cancer; self-stigma, the internalization of these negative attitudes; and experienced stigma, encompassing direct experiences of discrimination and exclusion [24, 26–28]. Stigmatization can lead to social isolation and multiple psychological consequences, including poor self-perception, loss of self-esteem, hopelessness, and depression [3, 29]. Perceiving oneself as “incomplete” or “worthless” in society can undermine one’s beliefs about life’s meaning and disrupt inner peace, thus diminishing spiritual well-being [30, 31]. Nevertheless, studies on the relationship between stigmatization and spiritual well-being have focused primarily on populations such as individuals living with HIV/AIDS and those with mental disorders [30, 32]. Cancer patients, who also experience high levels of stigma [25, 28, 33], remain underrepresented in this area of research.

According to Lazarus and Folkman’s stress and coping model [34–36], stress arises when individuals perceive an event as exceeding their coping resources. In this framework, illness-related uncertainty and stigma are cognitive stressors that can influence emotional responses and coping strategies, ultimately affecting outcomes such as spiritual well-being. Individuals with greater intolerance of uncertainty or heightened stigma may evaluate their illness as uncontrollable or threatening, thereby hindering their ability to maintain meaning, coherence, and spiritual peace [34–36]. The application of this model provides a theoretical lens for understanding how psychological and social stressors interact to shape spiritual adjustment in cancer patients.

In summary, although previous studies have emphasized the psychological burden of intolerance of uncertainty and stigmatization, the combined effects of these variables on the spiritual well-being of cancer patients have not been directly examined. Addressing this gap will contribute to a holistic understanding of the psychological, social, and existential challenges faced by cancer patients. Furthermore, identifying these factors will guide healthcare professionals—particularly nurses—in providing individualized, holistic, and spiritually sensitive care [25, 33, 37–39]. Accordingly, the aim of this study was to examine the relationship between attitudes toward cancer and intolerance of uncertainty with respect to spiritual well-being in cancer patients.

## Method

### Design

In the present study, a cross-sectional descriptive and correlational research design was used. The Strengthening the Reporting of Observational Studies in Epidemiology (STROBE) guidelines, which were developed to improve the reporting quality of observational studies, were used in the reporting of the studies [18, 40].

### Population and sample

The population of the study consisted of all patients diagnosed with cancer who were admitted to oncology outpatient clinics or who received inpatient treatment in two different public hospitals between March 1, 2024, and January 30, 2025. The minimum sample size to represent the population was calculated via the G*Power-3.1.9.2 program. Since no study directly addressing the relationship between attitudes toward cancer and intolerance of uncertainty and spiritual well-being in cancer patients has been conducted in the literature, a pilot study was conducted with 50 participants to calculate the required sample size. The calculation of the sample size was based on the correlation coefficient (*ρ* = 0.180) between spiritual well-being and the “impossibility of recovery” subdimension of attitudes toward cancer scale, which is in line with the results of the pilot study. On the basis of the 95% confidence level (1-α), 95% test power (1-β) and expected correlation coefficient *ρ* = 0.180, the minimum sample size to be reached in the study was calculated as 395 patients. A total of 430 patients were approached to participate in the study. Of these, 22 declined to participate, and 8 patients were excluded because of incomplete data, resulting in a final sample of 400 participants. All subsequent analyses were performed on these 400 complete cases. The study included a total of 400 patients. According to the post hoc power analysis, the statistical power of the test was calculated as 97% considering the 95% confidence level (1-α), the correlation coefficient *ρ* = 0.193, and the sample size of 400 people. Convenience sampling was used for sample selection in the study.

### Inclusion criteria

- Patients who were diagnosed with cancer (at least 6 months)
- Being informed about the disease
- Karnofsky performance index above 40
- Being able to communicate verbally (clear consciousness, no stuttering or speech impediment)
- Provide voluntary consent to participate in the study
- 18 years of age or older
- Being literate

### Exclusion criteria

- A history of a significant stressor or traumatic life event in the last 6 months, other than a cancer diagnosis
- A diagnosis of intellectual disability
- History of neurocognitive or psychotic disorders
- Incomplete or incomplete completion of the questionnaires

### Instruments

Information form: The form, developed by the researchers in line with the literature review, includes a total of 25 questions to evaluate the individual and disease-related characteristics of the patients [6, 8, 28].

#### Three-factor spiritual well-being scale (TFSWS)

Developed by Ekşi and Kardaş [12], the TFSWS was designed to measure the spiritual well-being levels of individuals. The scale has a 5-point Likert-type scale (scored from 1: not at all suitable for me to 5: completely suitable for me) and consists of 29 items in total. The score from the scale is calculated on the basis of the mean score of each subdimension and the sum of these means, and high scores indicate that the individual has a high level of spiritual well-being. The items belonging to the “anomie” subdimension are reverse scored when the total score is to be calculated. The scale, developed specifically for Turkish culture, has three subdimensions: transcendence (items 1, 4, 5, 8, 9, 12, 13, 16, 17, 20, 21, 24, 25, 27, and 29), harmony with nature (items 2, 6, 10, 14, 18, 22, and 28), and anomie (items 3, 7, 11, 15, 19, 23, and 26). A total score of 29–145 can be given from the scale. The Cronbach’s alpha internal consistency coefficients of the scale were 0.953 for the transcendence subdimension, 0.864 for the harmony with nature subdimension, 0.853 for the anomie subdimension, and 0.886 for the total scale [12, 41]. In this study, the Cronbach’s alpha internal consistency coefficients of the scale were calculated as 0.94, 0.85, 0.76, and 0.89 for transcendence, harmony with nature, anomie, and the total score, respectively. The TFSWS was chosen for this study because it was developed and validated within the Turkish cultural context and provides a comprehensive assessment of individuals’ spiritual well-being through three dimensions: transcendence, harmony with nature, and anomie. Although it has not been specifically validated among cancer patients, the scale’s conceptual framework aligns with the multidimensional nature of spirituality and has been effectively applied in diverse clinical and psychosocial contexts, supporting its suitability for use in oncology populations. In this study, spiritual well-being was operationally defined as the individual’s perceived sense of meaning, peace, and connectedness, as measured by the total score obtained from the TFSWS.

#### Questionnaire for the measurement of attitudes toward cancer (cancer label)-patient version (QMATC-PV)

Developed to assess attitudes toward cancer patients, the QMATC-PV was originally developed by Cho et al. in 2013 [39]. The scale was adapted into Turkish, and a validity and reliability study was conducted by Yılmaz et al. (2017), and the internal consistency coefficient (Cronbach’s alpha) was 0.88. The scale, which consists of a total of 12 items, uses a 4-point Likert-type rating system (1: Strongly disagree—4: Strongly agree). The scale consists of three subdimensions: “impossibility of recovery” (items 1–4), “labeling of cancer patients” (items 5–8) and “experiencing social discrimination” (items 9–12). The evaluation is based on the mean score of the items, and a mean score of 2.5 and above indicates that the individual has negative attitudes toward cancer and has a high level of stigmatization. There are no reverse items in the scale [26, 28, 42]. In this study, the internal consistency coefficient (Cronbach’s alpha) of the scale was calculated as 0.86. The QMATC-PV was selected for this study because it directly evaluates patients’ perceptions and stigmatizing attitudes toward cancer, which are conceptually aligned with the study’s aim of examining psychosocial and cognitive factors influencing spiritual well-being. Its validated Turkish version and concise structure make it suitable for use in clinical oncology settings. In this study, attitudes toward cancer were operationally defined as individuals’ cognitive and emotional evaluations of cancer patients, including their beliefs about recovery, labeling, and perceived discrimination. This construct was measured via the QMATC-PV, with higher mean scores indicating more negative attitudes and greater perceived stigma toward cancer.

#### Intolerance of uncertainty scale (IUS)

The short form of the IUS (IUS-12), developed to assess individuals’ reactions to uncertainty, was originally developed by Carleton, Norton, and Asmundson in 2007 [43]. The Turkish validity and reliability study of the scale was conducted by Sarıçam et al. The IUS consists of a total of 12 items and is administered with a 5-point Likert-type rating system (1: Not at all suitable for me–5: Completely suitable for me). The scale consists of two subdimensions: “prospective anxiety (items 1–7)” and “inhibitory anxiety (items 8–12).” The scale has no reverse items and no cutoff point. The score of the scale is calculated on the basis of the sum score. The range of points that can be calculated for the total scale is between 12 and 60. As the score obtained from the scale increases, the level of intolerance to uncertainty increases in the relevant subdimension and in the total score of the scale. Cronbach’s alpha coefficient of the scale was found to be 0.88 for the total scale, 0.84 for the prospective anxiety subdimension, and 0.77 for the inhibitory anxiety subdimension [15]. In this study, the internal consistency coefficients (Cronbach’s alpha) for prospective anxiety, inhibitory anxiety, and the total scale were calculated as 0.77, 0.88, and 0.88, respectively. The IUS-12 was chosen for this study because it provides a reliable and concise measure of individuals’ emotional and cognitive responses to uncertain or ambiguous situations, which is particularly relevant in understanding how uncertainty associated with a cancer diagnosis may affect psychological and spiritual well-being. In this study, intolerance of uncertainty was operationally defined as the tendency to react negatively at emotional, cognitive, and behavioral levels to uncertain events or situations, as measured by the total score of the IUS-12. Higher scores represent greater intolerance of uncertainty.

### Data collection

The data were collected by the researcher through face-to-face interviews between March 1, 2024, and January 30, 2025, after the approval of the ethics committee and the necessary permission from the relevant institutions. The data collection form lasted approximately 25–30 min.

### Analysis

The data were analyzed via the Statistical Package for the Social Sciences (SPSS) 23.0 program. The normality of the distribution of the data was evaluated via the Kolmogorov–Smirnov test. Categorical variables were defined as frequency (*n*) and percentage (%) values, and continuous variables were defined as the mean ± standard deviation (X̄ ± SS) and minimum and maximum values. Independent sample *t* tests were used for comparisons between two groups, and one-way analysis of variance (ANOVA) was used for more than two groups; significant differences between groups were analyzed with the Bonferroni correction. The relationships between spiritual well-being, attitudes toward cancer, intolerance of uncertainty, and continuous variables were analyzed via Pearson correlation analysis. All variables potentially affecting spiritual well-being were evaluated via multiple linear regression analysis (backward elimination method). Before the analyses were conducted, the data were screened for missing values and outliers. Cases with missing data were excluded via listwise deletion, and the analyses were performed on complete cases only. Outliers were identified through standardized *z* scores and the Mahalanobis distance, and observations exceeding ± 3 standard deviations were examined and removed when necessary to ensure that the assumptions of multivariate analysis were met. The statistical significance level was accepted as *p* < 0.05.

### Ethical issues

Ethical approval from Bandırma Onyedi Eylül University Health Sciences Non-Interventional Research Ethics Committee was obtained for the conduct of the study with the decision dated 16.01.2024 and numbered 2023-254. After ethics committee approval, institutional permission was obtained from Bursa Uludağ University Health Practice and Research Center Hospital (Date: 19.02.2024, Number: E-73115338-819-126164) and Bursa Ali Osman Sönmez Oncology Hospital (Date: 12.07.2024; Number: E-67508481-929-24859842). In addition, the aim of the study was explained to the patients, and written informed consent was obtained through the Consent Form. The study was conducted in accordance with the ethical principles of the Declaration of Helsinki.

## Results

Table 1 presents the comparison of the patients’ mean TFSWSs based on the distributions of their individual characteristics. Table 1 shows that 54.8% of the patients were female, with a mean age of 52.67 ± 14.67 years (range, 18–89). A total of 31% were high school graduates, 76% were married, 79.8% were unemployed, and 49.3% had an income equal to their expenses. Additionally, 85.3% lived with their families, 43.5% had previously smoked and quit, 67.8% had never consumed alcohol, 67% were fully independent, and 56.8% engaged in daily exercise.

**Table 1:** Individual characteristics of the patients and comparison of mean TFSWSs (*n* = 400)

Variables	Number (*n*)	Percentage (%)	*x̅*	± SS	*t*/*F*	*p*
**Gender**						
Male	181	45.3	117.28	16.61	*t* = -.489	*p* = 0.137
Female	219	54.8	119.74	16.34		
**Age**			52.67	14.67		
**Educational status**						
Primary school	109	27.3	120.21	13.98	*F* = 1.165	*p* = 0.326
Middle school	63	15.8	117.46	18.72		
High school	124	31.0	117.08	17.85		
University	89	22.3	118.60	15.71		
Postgraduate	15	3.8	125.00	15.73		
**Marital status**						
Married	304	76.0	119.08	16.81	*t* = 0.983	*p* = 0.326
Single/divorced/husband deceased	96	24.0	117.18	15.40		
**Employment status**						
Working	81	20.3	119.17	15.45	*t* = 0.331	*p* = 0.741
Not working	319	79.8	118.49	16.76		
**Income level**						
Income less than expenditure^a^	136	34.0	118.11	16.69	*F* = 0.109	*p* = 0.896
Income equal to expenditure^b^	197	49.3	118.97	16.16		
Income more than expenditure^c^	67	16.8	118.64	17.24		
**The person you live with**						
Family^a^	341	85.3	118.95	17.08	*F* = 1.683	*p* = 0.187
Alone^b^	28	7.0	113.21	14.19		
With children	31	7.8	119.96	9.81		
**Smoking status**						
Current smoker	67	16.8	116.40	15.97	*F* = 1.508	*p* = 0.223
Never smoked	159	39.8	120.25	15.12		
Former smoker	174	43.5	118.00	17.78		
**Alcohol use status**						
Current user^a^	25	6.3	111.88	18.44	*F* = 8.893	***p* < 0.001** ***b* > *c***
Never used^b^	271	67.8	120.95	15.39		
Former user^c^	104	26.0	114.19	17.53		
**Perception of general health status**						
Good	121	30.3	38.46	13.83	*F* = 0.096	*p* = 0.909
Moderate	213	53.3	38.75	15.65		
Poor	66	16.5				
**Level of independence**						
Fully independent^a^	268	67.0	118.41	16.68	*F* = 0.245	*p* = 0.783
Semidependent^b^	87	21.8	119.67	16.65		
Fully dependent^c^	45	11.3	117.86	15.18		
**Daily exercise**						
Yes	227	56.8	117.69	17.30	*t* = −.305	*p* = 0.193
No	173	43.3	119.86	15.31		

^a, b, c^Mean differences between groups. *F* one-way ANOVA, *t* independent sample *t* test; *p < 0.05; **p < 0.001

The physical and mental health characteristics of the patients are presented in Table 2. While 35% of the participants had comorbid physical illnesses, 65% did not. Hypertension was reported in 17% of the patients, diabetes in 14.2%, COPD and hyperthyroidism in 5%, and lumbar disc herniation in 1.8%. Data on other physical conditions are detailed in Table 2. Additionally, 2.8% of the patients had been diagnosed with a mental disorder prior to their cancer diagnosis, with the most common being major depression (1.3%), panic disorder (1.0%), anxiety disorder (0.3%), and bipolar disorder (0.3%). Following the cancer diagnosis, 5% of the patients were diagnosed with a mental disorder, most commonly anxiety disorder (1.8%), major depression (1.5%), sleep disorder (1.0%), or panic disorder (0.8%) (Table 2).

**Table 2:** Characteristics of patients’ physical and mental health and comparison of mean TFSWS scores (*n* = 400)

Variables	Number (*n*)	Percentage (%)	*x̅*	± SS	*t*/*F*	*p*
**Presence of comorbid physical illness**						
Present	140	35.0	118.25	17.25	*t* = − 0.338	*p* = 0.736
Absent	260	65.0	118.83	16.09		
**Hypertension**						
Present	68	17.0	121.60	18.04	*t* = 0.826	*p* = 0.409
Absent	332	83.0	118.47	16.41		
**Diabetes**						
Present	57	14.2	116.82	18.40	*t* = − 0.892	*p* = 0.373
Absent	343	85.8	118.93	16.16		
**Hormonetherapy**						
Yes	56	14.0	113.94	20.13	*t* = − 2.304	***p* = 0.022**
No	344	86.0	119.39	15.72		

^a, b, c^Mean differences between groups. *F* one-way ANOVA, *t* independent sample *t* test; *p < 0.05; **p < 0.001

The distribution of the cancer-related characteristics of the patients is presented in Appendix A and B. Among the participants, 8.5% reported that they did not know their type of cancer. The most common cancer types were breast cancer (22.0%), lung cancer (15.0%), colon cancer (11.0%), and pancreatic cancer (5.5%). Details on other cancer types are provided in Table 3. Additionally, 33.8% of the patients stated that they did not know their cancer stage. The proportion of patients with stage I disease was 10.5%, that with stage II disease was 12.8%, that with stage III disease was 15.0%, and that with stage IV disease was 28.0%. The mean duration of illness was 2.99 ± 3.05 years (range: 6 months to 25 years). Surgical treatment was given to 63.0% of the patients, hormone therapy was given to 14.0%, chemotherapy was given to 94.0%, and radiotherapy was given to 37.8%. The proportion of patients receiving oral chemotherapy was 17.0% (see Appendix A and B).

**Table 3:** Mean scores of the patients on the scales

Scales	*X*	± SD	Min	Max
**Spiritual well-being**	118.63	** ± **16.49	51.00	145.00
Transcendence	63.95	** ± **11.89	15.00	75.00
Harmony with nature	30.91	** ± **4.54	7.00	35.00
Anomie	18.24	** ± **6.07	7.00	35.00
**Attitudes toward cancer**	2.06	** ± **0.50	1.00	4.00
Impossibility of recovery	2.07	** ± **0.67	1.00	4.00
Labeling of cancer patients	2.37	** ± **0.57	1.00	4.00
Social discrimination	1.74	** ± **0.69	1.00	4.00
**Intolerance of uncertainty**	36.45	** ± **9.80	13.00	60.00
Prospective anxiety	22.11	** ± **5.60	7.00	35.00
Inhibitory anxiety	14.34	** ± **5.26	5.00	25.00

As shown in Table 1, the mean total score of the TFSWS significantly differed by alcohol use status (F(2, 397) = 8.89, *p* < 0.001, *η*^2^ = 0.04). Patients who never consumed alcohol had significantly higher spiritual well-being scores than did those who used alcohol and then quit alcohol (*p* < 0.001). Additionally, patients who had not received hormone therapy had significantly higher mean TFSWSs than those who had (*t*(398) = 2.30, *p* = 0.022, *d* = 0.30) (Table 3). No statistically significant differences were found between the mean TFSWS scores and other individual characteristics, physical and mental health variables, or cancer-related characteristics (*p* > 0.05) (see Appendix A and B).

Table 3 presents the mean scores of the patients on the TFSWS and its subdimensions. The mean total TFSWS score was 118.63 ± 16.49. The mean scores for the subdimensions of transcendence, harmony with nature, and anomie were 63.95 ± 11.89, 30.91 ± 4.54, and 18.24 ± 6.07, respectively. The mean total score for the QMATC-PV was 2.06 ± 0.50. The subdimension scores for impossibility of recovery, labeling of cancer patients, and experiencing social discrimination were 2.07 ± 0.67, 2.37 ± 0.57, and 1.74 ± 0.69, respectively. The mean total IUS score was 36.45 ± 9.80, with subdimension scores of 22.11 ± 5.60 for prospective anxiety and 14.34 ± 5.26 for inhibitory anxiety (Table 3).

The relationships between spiritual well-being and attitudes toward cancer, intolerance of uncertainty, and continuous variables are presented in Table 4. The findings indicate a weak but statistically significant negative correlation between the total mean score of spiritual well-being and the impossibility of recovery (*r* = –0.193, 95% CI [–0.296, –0.090], *p* < 0.001) and social discrimination (*r* = –0.213, 95% CI [–0.307, –0.123], *p* < 0.001) subdimensions. Additionally, a low-level significant negative correlation was found between spiritual well-being and inhibitory anxiety (*r* = –0.142, 95% CI [–0.237, –0.040], *p* < 0.05). However, no statistically significant correlations were detected between spiritual well-being and the labeling of cancer patients (*r* = –0.055, 95% CI [–0.178, 0.055], *p* = 0.277), prospective anxiety (*r* = –0.045, 95% CI [–0.146, 0.055], *p* = 0.369), age (*r* = 0.038, 95% CI [–0.050, 0.138], *p* = 0.447), or duration of illness (*r* = 0.019, 95% CI [–0.060, 0.083], *p* = 0.701) (Table 4).

**Table 4:** Correlations between spiritual well-being and attitudes toward cancer, intolerance of uncertainty and continuous variables (*n* = 400)

Variables		Spiritual well-being	95% CI	
			Lower	Upper
Impossibility of recovery	*r*	− 0.193	− 0.296	− 0.090
	*p*	0.000^**^		
Labeling of cancer patients	*r*	− 0.055	− 0.178	0.055
	*p*	0.277		
Social discrimination	*r*	− 0.213	− 0.307	− 0.123
	*p*	0.000^**^		
Prospective anxiety	*r*	− 0.045	− 0.146	0.055
	*p*	0.369		
Inhibitory anxiety	*r*	− 0.142	− 0.237	− 0.040
	*p*	**0.004**		
Age	*r*	0.038	− 0.050	0.138
	*p*	0.447		
Duration of illness	*r*	0.019	− 0.060	0.083
	*p*	0.701		

*r*, Pearson correlation coefficient. *p < 0.05, **p < 0.001

The results of the regression analysis conducted to identify the factors affecting the level of spiritual well-being of patients are presented in Table 5. The model explained 12% of the variance in spiritual well-being levels (*p* < 0.001) (Table 5).

**Table 5:** Factors affecting patients’ spiritual well-being (*n* = 400)

Variables	Standardize nonexistent coefficient		Standardize coefficient	*t*	*p*	95% CI	
	***B***	SE	Beta			Lower	Upper
(Constant)	130.251	4.417		29.488	0.000	121.567	138.935
Impossibility of recovery	− 4.139	1.591	− 0.169	− 2.602	0.010	− 7.267	− 1.011
Labeling	3.013	1.748	0.105	1.724	0.086	− 0.423	6.448
Social discrimination	− 3.920	1.236	− 0.165	− 3.173	0.002	− 6.350	− 1.491
Inhibitory anxiety	− 0.323	0.154	− 0.103	− 2.106	0.036	− 0.625	− 0.021
Alcohol use status—current drinker^a^	− 9.670	3.255	− 0.142	− 2.971	0.003	− 16.068	− 3.271
Alcohol use status—used and quit	− 7.752	1.792	− 0.206	− 4.327	0.000	− 11.274	− 4.229
Receiving hormone therapy—no^b^	4.538	2.259	0.096	2.009	0.045	0.096	8.980

*F*(1, 391) = 8.772, *p < 0.001; Adj. *R*^2^ = 0.120. a: reference category, never used; b: reference category, yes. Variables included in the regression model: impossibility of recovery, stigmatization, social discrimination, inhibitory anxiety, prospective anxiety, alcohol use status, and hormone treatment status

According to the results, a one-unit increase in the impossibility of recovery subdimension was associated with a 0.169-unit decrease in spiritual well-being (*β* = –0.169, 95% CI [–7.27, –1.01], *p* < 0.05). Similarly, a one-unit increase in *social discrimination* led to a 0.165-unit decrease in spiritual well-being (*β* = –0.165, 95% CI [–6.35, –1.49], *p* < 0.001). An increase of one unit in *inhibitory anxiety* was associated with a 0.103-unit decrease in spiritual well-being (*β* = –0.103, 95% CI [–0.65, –0.02], *p* < 0.05).

Regarding alcohol use, the spiritual well-being scores of current drinkers were significantly lower than those of individuals who had never consumed alcohol (*β* = –0.142, 95% CI [–16.07, –3.27], *p* < 0.01). Patients who had used alcohol in the past but had quit had spiritual well-being scores 0.206 units lower than those who had never consumed alcohol (*β* = –0.206, 95% CI [–11.274, –4.229], *p* < 0.001). Moreover, the spiritual well-being of patients who did not receive hormone therapy was 0.096 units higher than that of patients who did (*β* = 0.096, 95% CI [0.10, 8.98], *p* < 0.05).

## Discussion

### Spiritual well-being, attitudes toward cancer, and intolerance of uncertainty levels among cancer patients

Considering the minimum and maximum possible scores on the Spiritual Well-Being Scale, participants scored above the mean on the total scale and on the subdimensions of transcendence and harmony with nature. In contrast, they scored below the midpoint in the Anomie subdimension. In a study conducted by Turan and Dural [11] in Türkiye with cancer patients using a different measurement tool, the spiritual well-being level of the participants was found to be above the mean. Similarly, in a study conducted by Khiyali et al. [8] in Iran using a different measurement tool, cancer patients were reported to have moderate levels of religious and existential well-being as well as total spiritual well-being. In a study conducted in Türkiye with patients diagnosed with gastrointestinal cancer, Kavak, Özdemir, and Dural [44] used a different measurement tool and reported that the total spiritual well-being and faith levels of the participants were significantly above the mean. In the same study, the scores of the participants in the subdimensions of meaning and peace were reported to be slightly above the mean [44]. In addition, different studies have shown that cancer patients’ spiritual well-being is moderate or high [9, 10].

The fact that cancer patients’ total score on the spiritual well-being scale is above the mean indicates that these individuals generally experience high levels of spiritual well-being. In addition, the higher-than-mean scores on the transcendence and harmony with nature subscales indicate that patients tend to turn to spiritual resources and establish a deeper connection with nature. Life-threatening diseases such as cancer may lead individuals to search for spiritual meaning, and this process may contribute to the development of a deeper sense of awareness and harmony with life. However, low scores obtained from the anomie subdimension indicate a high level of anomie, indicating that the participants may have weakened ties with spiritual values and belief systems and may experience a lack of meaning, lack of direction, and a sense of emptiness. Social support systems, access to health services, and spiritual support may be important factors influencing these low anomie scores. In addition, cancer patients’ search for meaning and development of new perspectives on life during the treatment process may positively affect their spiritual well-being. While the higher-than-mean spiritual well-being levels of the cancer patients in this study are similar to those reported in some studies in the literature, other studies reported different results. These differences may be due to factors such as cultural and religious beliefs, individual coping mechanisms and psychosocial support systems. In societies that attach importance to religious and spiritual values, such as Türkiye, patients’ tendency to question the meaning of life and seek support from spiritual sources may be more pronounced. In addition, patients’ access to health services and the prevalence of spiritual counseling and psychosocial support programs may also affect their level of spiritual well-being. Differences in measurement tools, the methods used in the studies and the stages of the participants in the disease process may also be another reason for the differences in the results.

Considering the minimum and maximum possible scores on the Attitudes Toward Cancer Scale, participants’ total scores and scores on all subdimensions were below the scale’s cutoff point of 2.5. The highest mean score was observed for the labeling of the cancer patient subdimension (2.37), whereas the lowest score was for the experiencing social discrimination subdimension (1.74). In addition, the total score of the participants (2.06) and the scores the participants received in the subdimension of the impossibility of recovery (2.07) were similar to points. These results differ from the results of the study conducted by Yılmaz et al. [28] in Türkiye using the same measurement tool. In the study conducted by Yılmaz et al. [28], the total stigmatization score of cancer patients (2.7) and the scores they received from the subdimensions (labeling of cancer patients, 2.5; experiencing social discrimination, 2.9; impossibility of recovery, 2.7) were above the cutoff point of the scale. These results indicate that negative attitudes toward cancer are common in patients [28]. On the other hand, Xu et al. [25] reported that the stigmatization levels of cancer patients were lower than the mean in a study conducted in China that used a different measurement tool. Similarly, in another study conducted by Mi, Jin, Zheng, Cheng, and Zhang [27] in China, patients’ levels of total stigma, internalized shame, social isolation, social rejection, and financial insecurity were reported to be moderate. In addition, Andres et al. [33] reported that 39% of cancer patients experienced cancer-related stigma in a large-scale study from eight Asian countries. In the literature, different studies have revealed that cancer patients living in different countries experience low, moderate, and high levels of stigmatization [45–47].

The results obtained from the current study show that cancer patients’ perceptions of stigmatization are low and that their negative attitudes toward cancer are relatively negative. The relatively high score in the labeling of the cancer patient subdimension may suggest that patients occasionally experience labeling or prejudice. However, low scores on the experiencing social discrimination subscale may indicate that such experiences are not common or that more supportive attitudes toward cancer patients prevail in society. The low perception of the impossibility of recovery suggests that patients have a more positive and hopeful perspective on treatment. In the present study, cancer patients experienced low levels of stigmatization, but different results have been reported in the literature. These differences may be due to factors such as cultural and social attitudes, cancer awareness, and access to health services. The higher stigmatization scores reported in studies conducted in Türkiye may be due to the higher prevalence of prejudices or misperceptions about cancer in society. In contrast, the low level of stigma in this study may reflect the impact of positive interventions such as information campaigns, support groups, and psychosocial support services. In addition, the socioeconomic status, education level, and cancer-related experiences of the sample group in this study may also affect the perception of stigma. Cross-cultural differences and the individual coping mechanisms of patients may also be other reasons for the differences in these results.

Considering the minimum and maximum possible scores on the Intolerance of Uncertainty Scale, participants’ total scores, as well as their scores on the Prospective Anxiety and Inhibitory Anxiety subdimensions, were found to be moderate. Similar to the results of this study, Akkuş and Menekli [6] conducted a study with cancer patients in Türkiye using the same measurement tool and reported that the total intolerance of uncertainty, prospective anxiety, and inhibitory anxiety levels of the patients were greater than the mean. However, in a study conducted by Shen et al. [5] in China using a different measurement tool, cancer patients’ total intolerance of uncertainty, anticipatory behavior, inhibitory behavior, and anticipatory emotion levels were found to be higher than the mean. In the study of Shen et al. [5], the highest mean score was found in the anticipatory behavior subdimension, which evaluates the behaviors that occur due to fear of future events. Inhibitory behavior and anticipatory emotion scores were found to be close to each other and at similar levels [5]. On the other hand, in a study conducted by Eyni and Mousavi [47] with cancer patients in Iran, the total level of intolerance to uncertainty and the scores obtained from the incompatibility and anxiety subdimensions were found to be lower than the mean. Similarly, in another study conducted by Poshtan et al. [16] in Iran, cancer patients’ intolerance of uncertainty levels was found to be lower than the mean. In addition to these studies, various national and international studies have shown that cancer patients experience low, moderate, or high levels of intolerance of uncertainty [17–20, 48, 49].

The moderate level of the participants’ intolerance of uncertainty scores and the scores they scored in the prospective anxiety and inhibitory anxiety subdimensions indicate that individuals react relatively moderately to uncertainty and that their anxiety levels are not excessively high or low. The moderate scores of cancer patients on the overall intolerance of uncertainty scale and its subdimensions may be directly related to the uncertainty created by the disease process. The cancer diagnosis and treatment process may increase individuals’ feelings of anxiety and uncertainty about the future. In particular, the unpredictability of treatment outcomes and uncertainties about the course of the disease may cause patients to have moderate levels of anticipatory anxiety and inhibitory anxiety. However, the trust relationships that patients establish with healthcare teams, social support systems, and psychological support services during the treatment process may enable them to respond to uncertainty in a more balanced manner. Moreover, some patients may manage their anxiety by developing coping mechanisms to address this uncertainty. Therefore, these results may reflect both the effects of uncertainty caused by the disease and the adaptation processes that patients develop against this situation. In this study, cancer patients’ intolerance of uncertainty levels was moderate, whereas different results have been reported in the literature. These differences may be due to various factors, such as cultural differences, disease experience, and psychosocial support systems. The greater intolerance of uncertainty scores in some studies conducted in Türkiye may be related to regional differences, a lack of information about cancer, or negative health beliefs. On the other hand, in studies conducted in different cultures, such as China and Iran, patients’ lower or higher levels of intolerance of uncertainty may be explained by access to health services, psychological support opportunities, and individual coping strategies. In addition, the different measurement tools and methodological differences used in the studies may have contributed to the variability between these results.

### Relationships among uncertainty, stigma, and spiritual well-being

In this study, higher perceptions of the impossibility of recovery and experiences of social discrimination were associated with lower levels of spiritual well-being. These findings suggest that when patients lose hope for recovery or feel socially excluded, their sense of meaning, purpose, and spiritual connectedness may be undermined. However, no significant relationship was found between the labeling of cancer patients and their spiritual well-being. In the literature, no study has directly examined the relationship between attitudes toward cancer and the spiritual well-being of cancer patients. However, studies have examined the relationships between these concepts in different samples, such as individuals living with HIV/AIDS and individuals with mental disorders. In a study conducted by Hutson et al. [31] with individuals living with HIV/AIDS in the USA, a weak negative relationship between spiritual well-being and personalized stigma and negative self-image subdimensions was reported. Similarly, in a study conducted by Zarei et al. [32] with individuals living with HIV/AIDS in Iran, a weak negative relationship was found between spiritual beliefs and perceived stigma. In addition, in a study conducted by Taheri, Shamsaei, Tapak, and Sadeghian [30] with individuals with mental disorders, a moderate negative relationship between stigmatization and spiritual well-being was reported. In addition, Porter et al. [48] and Grodensky et al. [49] reported a negative relationship between stigma and spiritual well-being in older individuals living with HIV/AIDS.

Cancer patients may lose hope for recovery due to uncertainty in the treatment process and concerns about the course of the disease, which may reduce their spiritual well-being. Furthermore, patients who perceive exclusion or discrimination from their social environment may struggle more spiritually due to loneliness and a lack of social support. On the other hand, the lack of a significant relationship between the labeling of cancer patients and spiritual well-being may be explained by the fact that patients do not internalize such labeling or cope with these perceptions through spiritual beliefs and support mechanisms.

In the present study, higher levels of inhibitory anxiety were associated with lower spiritual well-being, whereas no significant relationship was found between prospective anxiety and spiritual well-being. Although no studies have directly examined the relationship between intolerance of uncertainty and spiritual well-being in cancer patients, studies have examined similar relationships in different samples, such as older adults and kidney transplant recipients. In a study conducted by Akgün Şahin, Deniz, Akça, Uymaz Aras, and Doğan [23] with elderly individuals living in the Eastern Anatolia Region of Türkiye during the COVID-19 period, a weak negative relationship was reported between spiritual well-being and inhibitory anxiety. In the same study, no significant relationship was found between prospective anxiety and intolerance of uncertainty total score and spiritual well-being. Similarly, in a different study conducted with elderly individuals in Iran, a moderate positive relationship was found between spiritual well-being and tolerance for uncertainty [50]. In addition, in a study conducted by Asif, Asad, Ahme, and Emmanuel [21] with individuals aged 55 years and over in Pakistan, a negative moderate relationship was found between spirituality and intolerance of uncertainty, prospective anxiety, and inhibitory anxiety. On the other hand, in a study conducted by Bakan [22] with kidney transplant recipients in Türkiye, no significant relationship was found between intolerance of uncertainty and spiritual well-being.

The negative association between inhibitory anxiety and spiritual well-being may be understood in light of behavioral inhibition theory, which suggests that individuals experiencing high inhibitory anxiety tend to withdraw or “freeze” in the face of uncertainty, limiting their ability to take action, make meaning, and use adaptive coping strategies. For cancer patients, this form of anxiety may hinder their engagement with spiritual resources, prayer, or meaning-making processes that typically foster resilience and hope. In contrast, prospective anxiety—which reflects anticipatory concern about future events—does not necessarily impede spiritual well-being. Patients who maintain hope through faith or social support may even channel such anticipatory worry into proactive coping and reliance on spiritual beliefs. Accordingly, inhibitory anxiety seems to suppress spiritual well-being by promoting avoidance and emotional disengagement, whereas prospective anxiety may coexist with constructive adaptation. These findings highlight that distinct dimensions of uncertainty-related anxiety influence spiritual well-being through different psychological pathways.

In this study, patients who currently used or had previously used alcohol reported lower levels of spiritual well-being than did those who had never consumed alcohol. Unlike the results of the study, in the study conducted by Zhou et al. [51] with cancer patients, individuals who consumed alcohol were found to have lower spiritual care needs. In a study conducted by Churakova, Burlaka, and Parker [52] with adults in the USA, a negative moderate relationship was found between alcohol consumption and spiritual well-being. In addition, studies have shown a negative relationship between alcohol consumption and spiritual well-being in various samples, such as adolescents and university students [53, 54]. Alcohol use may negatively affect patients’ orientation toward spiritual resources. Cancer patients may seek spiritual support while coping with health-related uncertainties and stress. However, alcohol use may function as an emotional avoidance and coping behavior. Alcohol use can also make it difficult to recognize and meet spiritual needs. In contrast, patients who do not drink alcohol may often develop healthier coping strategies and may be able to maintain their spiritual well-being by making greater use of spiritual support resources. In the literature, studies have evaluated the relationships between alcohol consumption and spiritual care needs and spiritual well-being in different ways. These differences may be due to factors such as sample characteristics, cultural differences, and the psychosocial effects of alcohol use on individuals.

In this study, patients who did not receive hormone therapy reported slightly greater levels of spiritual well-being than did those who underwent hormone therapy. When the current literature was reviewed, no study examining the relationship between the type of treatment received and spiritual well-being in cancer patients was found. This result may be explained by the physical and psychological side effects of hormone therapy, which may negatively affect the spiritual well-being of patients. Hormone therapy may cause symptoms such as fatigue, mood changes, and anxiety, making patients’ spiritual support difficult to find [55]. Moreover, uncertainties and concerns about the treatment process may affect patients’ search for spiritual balance and meaning. On the other hand, individuals who do not receive treatment may feel better physically, which may contribute to the higher perception of spiritual well-being levels of patients who do not use alcohol.

## Limitations

This study has several limitations. First, its cross-sectional design restricts causal inferences, while associations between intolerance of uncertainty, attitudes toward cancer, and spiritual well-being were identified; the directionality of these relationships cannot be determined. Second, the use of self-report measures may introduce response bias and social desirability effects. Third, the study was conducted with patients from two public hospitals, which may limit the generalizability of the findings to other settings or cultural contexts. Finally, factors such as disease stage, type of treatment, or religious/spiritual background were not controlled for, which may have influenced spiritual well-being levels. Although the model explained 12% of the variance in spiritual well-being, this relatively low explained variance suggests that other unmeasured factors may also play important roles. Variables such as religious coping, social and family support, health literacy, personality traits, and the degree of meaning-making might further influence patients’ spiritual well-being. Future studies could incorporate these variables to develop a more comprehensive understanding of the psychosocial and spiritual determinants of well-being among cancer patients.

## Conclusion

In this study, individuals diagnosed with cancer demonstrated spiritual well-being levels above the mean, whereas their attitudes toward cancer were generally negative and their intolerance of uncertainty was moderate. Among the subdimensions, labeling had the highest scores, whereas social discrimination and perceptions of the impossibility of recovery were particularly associated with lower spiritual well-being. Inhibitory anxiety has also emerged as a psychological factor that negatively influences patients’ spiritual well-being. Additionally, alcohol use and hormone therapy status appeared to be relevant factors associated with reduced spiritual well-being. These findings should be interpreted with caution, as they reflect the experiences of a specific sample within a particular cultural and clinical context. Nonetheless, they underscore the importance of addressing uncertainty intolerance and cancer-related stigma to promote spiritual well-being among cancer patients. Interventions focused on reducing inhibitory anxiety, along with patient education and comprehensive psychosocial and spiritual support programs, may enhance patients’ overall well-being. Healthcare providers, particularly nurses, psychologists, and spiritual counselors, play a vital role in recognizing these psychosocial challenges and implementing tailored interventions to support patients’ emotional and spiritual adaptation during the cancer journey.

## Implications for practice

To increase the spiritual well-being of cancer patients, psychosocial support programs (e.g., individual counseling, group therapies, and spiritual counseling) should be developed and implemented to help patients cope with the uncertainty and inhibitory anxiety they experience during the disease process. For patients with high levels of social discrimination and perceived impossibility of recovery, awareness-raising training and community-based support programs to promote stigma coping should be developed. For patients with elevated inhibitory anxiety, evidence-based interventions such as stress management and cognitive–behavioral therapy should be implemented to support their ability to manage uncertainty. In clinical practice, nurses and psycho-oncology professionals can play a key role by routinely assessing patients’ experiences of stigma and intolerance of uncertainty via brief screening tools or structured interviews during follow-up visits. Incorporating these assessments into routine care can help identify individuals at greater risk of spiritual distress, enabling timely referrals for psychosocial or spiritual counseling. Furthermore, interdisciplinary collaboration between nurses, psychologists, and spiritual care providers may enhance holistic support and improve overall well-being among cancer patients.

## Supplementary Information

Below is the link to the electronic supplementary material.

ESM 1 (DOCX 22.1 KB)

## Data Availability

No datasets were generated or analysed during the current study
