# Supplementary material for: Intolerance of uncertainty and attitudes toward cancer as predictors of spiritual well-being in patients with cancer
Source: Support Care Cancer. 2026 Mar 27;34(4):375. doi: 10.1007/s00520-026-10606-0 (PMC13021687; doi:10.1007/s00520-026-10606-0)
Supplement: Supplementary file 1 — (DOCX 22.1 KB) [file 520_2026_10606_MOESM1_ESM.docx]

**Appendix A. Characteristics of patients’ physical and mental health and comparison of mean TFSWS scores** **(n=400)**

| **Variables** | **Number(n)** | **Percentage (%)** | **x̅** | **±SS** | **t/F** | **p** |
| --- | --- | --- | --- | --- | --- | --- |
| **Coronary artery disease** |  |  |  |  |  |  |
| Present | 15 | 3,8 | 111,86 | 16,16 |  |  |
| Absent | 385 | 96,3 | 118,89 | 16,46 |  |  |
| **COPD** |  |  |  |  |  |  |
| Present | 20 | 5,0 | 112,10 | 19,03 |  |  |
| Absent | 380 | 95,0 | 118,97 | 16,30 |  |  |
| **Hyperthyroidism** |  |  |  |  |  |  |
| Present | 20 | 5,0 | 121,60 | 18,04 |  |  |
| Absent | 380 | 95,0 | 118,47 | 16,41 |  |  |
| **Hepatitis** |  |  |  |  |  |  |
| Present | 3 | ,8 | 118,66 | 17,61 |  |  |
| Absent | 397 | 99,3 | 118,62 | 16,50 |  |  |
| **Claudication** |  |  |  |  |  |  |
| Present | 1 | ,3 | 140,00 | . |  |  |
| Absent | 399 | 99,8 | 118,57 | 16,47 |  |  |
| **Asthma** |  |  |  |  |  |  |
| Present | 4 | 1,0 | 115,25 | 16,17 |  |  |
| Absent | 396 | 99,0 | 118,66 | 16,51 |  |  |
| **Lumbar disc herniation** |  |  |  |  |  |  |
| Present | 7 | 1,8 | 123,28 | 11,94 |  |  |
| Absent | 393 | 98,3 | 118,54 | 16,56 |  |  |
| **Epilepsy** |  |  |  |  |  |  |
| Present | 1 | ,3 | 109,00 | . |  |  |
| Absent | 399 | 99,8 | 118,61 | 16,50 |  |  |
| **Rheumatoid arthritis** |  |  |  |  |  |  |
| Present | 1 | ,3 | 140,00 | . |  |  |
| Absent | 399 | 99,8 | 118,57 | 16,47 |  |  |
| **Hip dislocation** |  |  |  |  |  |  |
| Present | 1 | ,3 | 108,00 | . |  |  |
| Absent | 399 | 99,8 | 118,65 | 16,50 |  |  |
| **Kidney failure** |  |  |  |  |  |  |
| Present | 3 | ,8 | 113,66 | 11,01 |  |  |
| Absent | 397 | 99,3 | 118,66 | 16,52 |  |  |

Note: a,b,c: Indicates mean differences between groups. F: One-way ANOVA test; t: Independent sample t test; *: p<0.05; **: p<0.001

**Appendix A. Characteristics of patients’ physical and mental health and comparison of mean TFSWS scores** **(n=400) (Continued)**

| **Variables** | **Number(n)** | **Percentage (%)** | **x̅** | **±SS** | **t/F** | **p** |
| --- | --- | --- | --- | --- | --- | --- |
| **Vertigo** |  |  |  |  |  |  |
| Present | 1 | ,3 | 124,00 | . |  |  |
| Absent | 399 | 99,8 | 118,61 | 16,50 |  |  |
| **CMT disease** |  |  |  |  |  |  |
| Present | 1 | ,3 | 123,00 | . |  |  |
| Absent | 399 | 99,8 | 118,61 | 16,51 |  |  |
| **Arrhythmia** |  |  |  |  |  |  |
| Present | 2 | ,5 | 137,00 | 2,82 |  |  |
| Absent | 398 | 99,5 | 118,53 | 16,48 |  |  |
| **Prostate** |  |  |  |  |  |  |
| Present | 1 | ,3 | 109,00 | . |  |  |
| Absent | 399 | 99,8 | 118,65 | 16,50 |  |  |
| **Fibromyalgia** |  |  |  |  |  |  |
| Present | 2 | ,5 | 122,50 | 20,50 |  |  |
| Absent | 398 | 99,5 | 118,61 | 16,49 |  |  |
| **Behcet's disease** |  |  |  |  |  |  |
| Present | 1 | ,3 | 99,00 | . |  |  |
| Absent | 399 | 99,8 | 118,67 | 16,48 |  |  |
| **Hypercholesterol** |  |  |  |  |  |  |
| Present | 1 | ,3 | 126,00 | . |  |  |
| Absent | 399 | 99,8 | 118,61 | 16,50 |  |  |
| **Mental disorders before cancer diagnosis** |  |  |  |  |  |  |
| No | 389 | 97,3 | 118,65 | 16,60 | t=0.184 | p=0.854 |
| Yes | 11 | 2,8 | 117,72 | 12,48 |  |  |
| **Type** |  |  |  |  |  |  |
| Anxiety disorder | 1 | ,3 |  |  |  |  |
| Major depression | 5 | 1,3 |  |  |  |  |
| Bipolar disorder | 1 | ,3 |  |  |  |  |
| Panic Disorder | 4 | 1,0 |  |  |  |  |
| **Mental disorders during cancer diagnosis** |  |  |  |  |  |  |
| No | 380 | 95,0 | 118,74 | 16,63 | t=0.592 | p=0.554 |
| Yes | 20 | 5,0 | 116,50 | 13,61 |  |  |
| **Type** |  |  |  |  |  |  |
| Anxiety disorder | 7 | 1,8 |  |  |  |  |
| Major depression | 6 | 1,5 |  |  |  |  |
| Sleep disturbance | 4 | 1,0 |  |  |  |  |
| Panic Disorder | 3 | ,8 |  |  |  |  |
| **History of stress in the last 6 months** |  |  |  |  |  |  |
| No | 400 | 100,0 |  |  |  |  |

Note: a,b,c: Indicates mean differences between groups. F: One-way ANOVA test; t: Independent sample t test; *: p<0.05; **: p<0.001, CMT disease: Charcot-Marie-Tooth Disease

**Appendix B. Characteristics of the cancer-related variables and comparison of patients’ mean TFSWS scores (n=400)**

| **Variables** | **Number(n)** | **Percentage (%)** | **x̅** | **±SS** | **t/F** | **p** |
| --- | --- | --- | --- | --- | --- | --- |
| **Cancer diagnosis**  Unknown  Over  Prostate  Lung  Breast  Pancreas  Endometrium  Liver  Colon  Esophagus  Stomach  Tongue root  Kidney  Liposarcoma (soft tissue)  Melanoma  Testis  Palate  Brain  Bile  Thymic Carcinoma  Rectum  Multiple Myeloma  Lymphoma  Nasopharynx  Orbital  Thyroid  Larynx  Leukemia | 34  18  13  60  88  22  13  19  44  5  23  1  6  9  6  11  1  1  3  1  1  5  10  1  1  1  2  1 | 8,5  4,5  3,3  15,0  22,0  5,5  3,3  4,8  11,0  1,3  5,8  ,3  1,5  2,3  1,5  2,8  ,3  ,3  ,8  ,3  ,3  1,3  2,5  ,3  ,3  ,3  ,5  ,3 |  |  |  |  |
| **Disease stage** |  |  |  |  |  |  |
| Unknown | 135 | 33,8 | 119,96 | 13,58 | F=1.514 | p=0.197 |
| Phase 1 | 42 | 10,5 | 117,78 | 17,37 |  |  |
| Phase 2 | 51 | 12,8 | 116,00 | 19,16 |  |  |
| Phase 3 | 60 | 15,0 | 115,25 | 18,44 |  |  |
| Phase 4 | 112 | 28,0 | 120,34 | 16,80 |  |  |
| **Duration of illness (years)** |  |  | 2,99 | 3,05 |  |  |
| **Surgical treatment** |  |  |  |  |  |  |
| Yes | 252 | 63,0 | 118,96 | 15,94 | t=0.535 | p=0.593 |
| No | 148 | 37,0 | 118,05 | 17,41 |  |  |
| **Chemotherapy** |  |  |  |  |  |  |
| Yes | 376 | 94,0 | 118,83 | 16,25 | t=0.997 | p=0.319 |
| No | 24 | 6,0 | 115,37 | 19,93 |  |  |
| **Radiotherapy** |  |  |  |  |  |  |
| Yes | 151 | 37,8 | 119,00 | 16,31 | t=0.349 | p=0.727 |
| No | 249 | 62,3 | 118,40 | 16,62 |  |  |
| **Oral chemotherapy** |  |  |  |  |  |  |
| Yes | 68 | 17,0 | 116,50 | 19,24 | t=-1.170 | p=0.243 |
| No | 332 | 83,0 | 119,06 | 15,86 |  |  |

Note: a,b,c: Indicates mean differences between groups. T: t test. F: one-way ANOVA.
